# How Healthy Is It to Fortify Cocoa-Based Products with Cocoa Flavanols? A Comprehensive Review

**DOI:** 10.3390/antiox12071376

**Published:** 2023-07-03

**Authors:** Marta Palma-Morales, Sonia Melgar-Locatelli, Estela Castilla-Ortega, Celia Rodríguez-Pérez

**Affiliations:** 1 Biomedical Research Centre, Institute of Nutrition and Food Technology (INYTA) ‘José Mataix’, University of Granada, Avda. del Conocimiento s/n, 18071 Granada, Spain; martapalm@ugr.es; 2 Department of Nutrition and Food Science, Faculty of Pharmacy, University of Granada, Cartuja Campus, 18011 Granada, Spain; 3 Biomedical Research Instute of Malaga and Platform in Nanomedicine-IBIMA Platform BIONAND, 29590 Málaga, Spain; soniaml1998@uma.es (S.M.-L.); estela.castilla@ibima.eu (E.C.-O.); 4 Departament of Psychobiology and Methodology of Behavioural Sciences, Faculty of Psychology, University of Malaga, 29010 Málaga, Spain; 5 Instituto de Investigación Biosanitaria ibs.GRANADA, 18012 Granada, Spain

**Keywords:** cocoa, flavanols, fortified, human health, cardiovascular health, cognitive function, antioxidant activity

## Abstract

(1) Background: Cocoa’s healthy benefits may be attributed to the potent antioxidant activity of cocoa polyphenols, mainly flavanols, which have been characterised as existing in a high concentration in cocoa. However, the phenolic composition of cocoa and cocoa-derived products is highly variable, and manufacturing processes might significantly reduce their phenolic content. For that reason, the full characterisation of cocoa and cocoa-derived products before evaluating their bioactivity is crucial. The aim of this review is to analyse the available evidence on the effect of flavanol-fortified cocoa-derived products on human health. (2) Methods: Forty-eight clinical trials focused on the health effect of consuming flavanol-fortified drinks, bars and chocolate have been reviewed, with a total of 1523 subjects. (3) Results: Although studies differ widely in methodology, dosage, duration, and target population, beneficial effects of flavanol-rich cocoa consumption have been observed at doses ranging from 45.3 mg/d to 1078 mg/d, especially on cardiovascular health and cognitive function. (4) Conclusions: Considering the high consumption and acceptability of cocoa and cocoa-derived products, the fortification of cocoa products as well as other highly consumed foods with cocoa flavanols could be an effective strategy for health promotion.

## 1. Introduction

Cocoa is extracted from cocoa beans that are the fatty seeds of the *Theobroma cacao* tree. For consumption, the *Theobroma cacao* beans are processed as a paste (‘cocoa liquor’) containing non-fat cocoa solids and cocoa butter. ‘Cocoa powder’ results from eliminating cocoa butter from the liquor. In contrast, ‘chocolate’ combines cocoa liquor with additional cocoa butter and sugar; it is frequently enhanced with other components such as nuts or milk [1]. In Europe, the average per capita chocolate consumption reached 5 kg per year in 2022, and it is expected to grow at an average annual rate of around 4.8% between 2022 and 2027 [2]. Cocoa is rich in phenolic compounds (PCs), with the highest content of these being present in pure cocoa powder, followed by baking chocolate and dark chocolate; the lowest polyphenol content is found in so-called “white chocolate”, which is made from the cocoa butter [3,4].

PCs are considered essential in our diet. They are classified into various groups according to their chemical structure, with phenolic acids and flavonoids being the most abundant phenolic compounds in diet. Numerous studies have evaluated their effects on human health, attributing to them an important role in protecting the organism against external stimuli and in the elimination of reactive oxygen species [5]. PCs are synthetised by plants under normal and stress conditions and have various functions such as attracting insects for pollination and protecting against pathogens and ultraviolet radiation [6]. Thus, these compounds are present in foods such as tea, cocoa, fruits, vegetables, and honey, and their content varies widely depending on the variety, plant origin, agronomic and storage conditions, harvesting time, and climate, among other factors [7]. Improvements in lipid profiles, blood pressure, insulin resistance and systemic inflammation have been observed following consumption of PCs-rich foods, and these improvements are associated with improved cardiovascular health. In the central nervous system, these compounds counteract chronic and acute inflammatory processes, possess neuroprotective activity, and effectively reduce some signs and symptoms of neurodegenerative conditions, thus contributing to maintaining good brain health and overall quality of life [5]. 

**Figure 1:**
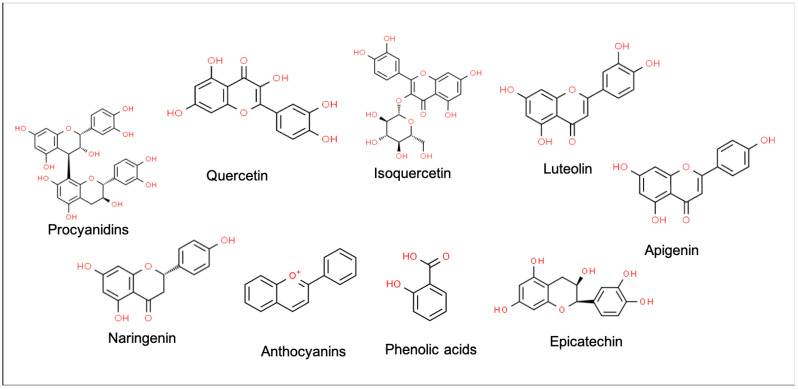
Main phenolic compounds of cocoa.

The long-recognized health benefits of cocoa may be attributed to the potent antioxidant activity of cocoa polyphenols, mainly flavanols, which are found in cocoa in a higher concentration (460–610 mg/kg of flavanol monomers; 4–5 g/kg of flavanol polymers) than in other plant-derived foods such as beans, apricots, blackberries, apples, and tea leaves [8]. The main flavonoids contained in cocoa are flavan-3-ols and their oligomers and polymers (procyanidins). In addition, cocoa contains flavonoids such as epicatechin, quercetin and isoquercetin; flavones such as luteolin and apigenin; flavanones such as naringenin; anthocyanins and phenolic acids (Figure 1). These compounds are highly related to antioxidant activity [9]. Specifically, cocoa flavanols benefit the cardiovascular system, have anti-inflammatory properties, reduce insulin resistance and enhance the growth of beneficial gut microbiota [1,3,10,11]. In addition, cocoa consumption has numerous health benefits that potentiate cognitive function, although the actions of cocoa on the nervous system have scarcely been investigated [12]. Since 2014, it has been possible to claim that ‘cocoa flavanols help maintain the elasticity of blood vessels, which contributes to normal blood flow’ [13]. Nevertheless, it is important to note that the phenolic composition of cocoa and cocoa-derived products is highly variable, and their contents in foods can be influenced by factors such as the genotype of the cocoa plant (Forastero/Amazónico, Criollo or Trinitario), the region, the method of cultivation, and the manufacturing processes (fermentation, drying, roasting, and particularly alkalizing, which decreases the phenolic content) [11,14,15]. According to other published studies, the decrease in phenolic compounds is 2–4 g/100 g dry weight, as raw cocoa nibs contain 6–8 g/100 g dry weight and cocoa powder contains 4 g/100 g dry weight [16]. In addition, the roasting process results in the epimerisation of (−)-epicatechin to (−)-catechin, and the epimerisation of (+)-catechin to (−)-catechin, and alkalinization also increases the levels of (−)-catechin, which is absorbed more poorly than the (+)-enantiomer. Therefore, both cocoa and chocolate contain mainly (−)-epicatechin and a large amount of (−)-catechin (which is less fully absorbed), while the concentration of (+)-catechin is very low in contrast to raw cocoa beans [16].

In view of the above, the objective of this work is to review the effects of consuming cocoa-based products fortified in cocoa flavanol on human health, in order to determine whether their manufacture may be advisable for health promotion.

## 2. Materials and Methods

The US National Library of Medicine National Institutes of Health (PubMed) was used to search the literature from 2000 to 2023, in order to gain an overview of all the available evidence of the effect of cocoa flavanol-fortified products on human health. The following search equation strategy was used: “flavanol-rich cocoa” [All Fields] OR “flavonoid-rich cocoa” [All Fields] OR “high-flavanol cocoa” [All Fields]. Medical subject heading (MeSH) terms were included to increase the power of the search. 

The main criteria of PICO (population, intervention, comparison, outcome) were followed to frame and answer the related clinical question. In this regard, the population included humans, the intervention was consumption of high-flavanol cocoa products, the comparison was made vs. low-flavanol cocoa products or placebo, and the outcomes were antioxidant effects, effects on cardiovascular health, visual function, cognitive function, and fatigability.

The inclusion criteria were the following: (1) clinical trials; (2) studies with cocoa or cocoa-derived products with a high flavanol content; (3) studies written in English. The exclusion criteria were (1) review articles; (2) studies written in languages other than English; (3) studies in animals; (4) studies without full access; (5) studies with the dose not made available; (6) studies with flavanol extracts; and (7) studies with combined treatments. Once the articles were selected based on reading the title, abstract and full text, and the results were classified according to the different health effects attributed to products enriched with cocoa flavanols, i.e., their effects on physical endurance and oxidative stress, cardiovascular risk factors, visual and cognitive function, and other effects in both healthy subjects and subjects with disease. These data have been summarised in different tables throughout the text, and a narrative review of the main outcomes has also been included.

## 3. Results

Forty-eight published articles were analysed, including a total of 1523 subjects with a mean age of 45.1 ± 16.6 years. Of the 1523 subjects, at least 762 were women (one study did not specify). The studies included different population groups (healthy subjects, subjects with renal disease, overweight or obese subjects, diabetic subjects, subjects with chronic heart failure, subjects with coronary artery disease, subjects with hypercholesterolaemia or hypertension, subjects with Parkinson’s disease, and subjects with multiple sclerosis), and included three different types of cocoa products: high-flavanol (HF) drinks, bars, and chocolate.

### 3.1. Effects in Healthy Subjects

Table 1 shows the effects of HF cocoa on exercise performance and oxidative stress in healthy subjects. A study conducted with 44 male endurance athletes reported a significant increase from baseline in the plasma ratio of follistatin/myostatin by modifying the levels of follistatin. Follistatin promotes adipose tissue browning and decreases body fat levels, and counteracts the myostatin blockade of muscle growth, making it an indicator of improved muscle function. Moreover, a decrease in body fat after 10 weeks of flavanol-rich cocoa consumption (425 mg flavanols/day) was observed. However, this may have led to a decrease in leptin levels (the hormone secreted by adipose tissue that sends the satiety signal to the brain, promotes lipolysis and depresses lipogenesis) [17]. In contrast, Patel et al. [18] observed no effects on exercise performance after a single dose of a chocolate bar containing from 88 to 1060 mg of flavanols in a study involving 15 healthy subjects aged 30 years on average. On the other hand, consumption of HF cocoa containing 425 mg of flavanols for 10 weeks reduced oxidative stress in a study with 56 male endurance athletes; however, no effects on aerobic capacity or exercise performance were observed [19]. Another study with 20 healthy men also reported a significant decrease in oxidative stress after a single dose of an HF cocoa drink (containing 187 mg of flavanols) compared to a control drink (14 mg of flavanols) [20]. Similarly, the study developed by Zhu et al. in 2005 showed a significant reduction in the susceptibility of erythrocytes to free radical-induced haemolysis, after consumption of 12.5–25 mg flavanols/kg body weight contained in cocoa beverages, compared to baseline [21].

Table 2 shows the effects of HF cocoa consumption by healthy individuals on cardiovascular risk factors. In a study conducted in middle-aged and elderly people (55–90 years), a significant improvement in several cardiovascular risk factors was observed, including a reduction in blood glucose and plasma triglycerides, as well as an increase in high-density lipoprotein (HDL) levels, physical performance, skeletal mass index, and quality of life after 12 weeks of consumption of an HF cocoa drink compared to placebo [22]. Similarly, consumption of flavanol-rich soluble cocoa for 4 weeks significantly increased HDL levels in healthy and moderately hypercholesterolemic subjects compared to milk consumption; however, a significant decrease in IL-10 from baseline was also observed [23].

A single dose of a chocolate bar containing 520 mg of flavanols significantly reduced pulse pressure, systolic blood pressure (SBP) and diastolic blood pressure (DBP) and attenuated the increase in pulse wave velocity in healthy sleep-deprived subjects (25.3 ± 3.6 years), as well as increased flow-mediated dilatation (FMD) and improved working memory accuracy compared to a flavanol-poor chocolate bar [24]. Likewise, a single dose of an HF cocoa drink significantly increased plasma levels of flavanols and FMD in healthy women (329 mg of flavanols) [25] and smokers (176–185 mg of flavanols) [26] compared to low-flavanol (LF) drinks. Brachial artery FMD also increased significantly from baseline after one week of consumption of an HF (918 mg) cocoa drink by male smokers aged 27 years [27]. Another study carried out in African Americans and Caucasian Americans showed a significant improvement in microvascular function and nitric oxide (NO) bioavailability, only in African Americans subjects, after a single dose of a cocoa drink containing 247.2 mg of flavanols [28]. A single dose of an HF cocoa drink (897 mg of flavanols) also showed platelet-modulating effects by reducing epinephrine-stimulated platelet activation and function from baseline, although to a lesser extent than aspirin, in healthy subjects from 22 to 49 years of age [29]. 

Chronic consumption of HF cocoa has also been shown to affect vascular function. Brachial artery FMD and plasma epicatechin significantly increased in a study conducted in healthy subjects after 2 weeks of consuming an HF chocolate bar [30]. Consumption of a cocoa beverage containing 821 mg of flavanols for 5 days increased FMD after hyperaemia in 48-year-old subjects [31], and increased NO-dependent vasodilation to ischaemia in healthy 44-year-old subjects [32]. However, it had no effects on blood pressure (BP). Consumption of HF (750 mg) dark chocolate for 8 weeks also had no effect on BP in prehypertensive healthy subjects (52.6 ± 12.6 years) [33].

**Table 2:** Effects on cardiovascular risk factors in healthy subjects.

Dose	Duration	Subjects	Effects	Ref
HF (563 mg) or LF (38 mg) cocoa drink	Single dose	10 healthy and physically active men 22.6 ± 0.3 years	↑** blood glucose pre-exercise	[34]
HF (179 mg), non-flavanol containing cocoa drink or placebo	12 weeks	61 healthy, middle-aged and elderly subjects (13 males, 48 females) 75.9 ± 5.8 years	↓** glycaemia ↓** TG ↑** HDL ↑** physical performance ↑** skeletal mass index ↑** quality of life	[22]
HF soluble cocoa (45.3 mg) or milk	4 weeks	24 healthy (11 males, 13 females) and 20 moderately hypercholesterolemic (9 males, 11 females) subjects 28 ± 8 years	↑** HDL ↑* dietary carbohydrate, protein and fibre intake ↓* IL-10	[23]
HF (520 mg) or LF (88.5 mg) chocolate bar Cross-over	Single dose	32 healthy sleep-deprived subjects (16 males, 16 females) 25.3 ± 3.6 years	↓** pulse pressure, SBP and DBP ↑** FMD ↓** increase in pulse wave velocity ↑** working memory accuracy	[24]
HF (329 mg) or LF (27 mg) cocoa drink	Single dose	10 healthy women 18–65 years	↑** FMD and oxygen saturation ↑** plasma epicatechin	[25]
HF (176–185 mg) or LF (<11 mg) cocoa drink Cross-over	Single dose	11 smokers (6 males, 5 females) 31 ± 1 years	↑** plasma levels of flavanols ↑** plasma levels of NO ↑** FMD	[26]
HF (918 mg) cocoa drink	1 week	6 male smokers 27 ± 1 years	↑* flow-mediated dilation of brachial artery	[27]
HF (247.2 mg/d) cocoa drink or non-flavanol containing drink Cross-over	Single dose	7 African American and 7 Caucasian American healthy subjects (8 males, 6 females) 22 ± 4 years	↑^a^ microvascular function ↑^a^ NO contribution	[28]
HF cocoa drink (897 mg) or aspirin (81 mg)	Single dose	16 healthy adults (8 males, 8 females) 22–49 years	↓* epinephrine-stimulated platelet activation and function	[29]
HF (259 mg) or LF (47.6) chocolate bar	2 weeks	22 healthy subjects (11 males, 11 females) 32.2 ± 3.1 years	↑** brachial artery FMD ↑** plasma epicatechin	[30]
HF cocoa drink (821 mg)	5 days	34 healthy subjects (13 males, 21 females) 47.9 ± 3.0 years	↑* FMD after hyperaemia ↑ pulse wave amplitude No effects on BP	[31]
HF (821 mg) cocoa drink or LF control drink	5 days	27 healthy subjects (11 males, 16 females) 44 ± 3.4 years	↑** pulse wave amplitude ↑** vasodilator response to ischaemia No effects on BP	[32]
HF dark chocolate (750 mg), tomato extract capsule (15 mg lycopene), or placebo Cross-over	8 weeks	36 prehypertensive healthy subjects (19 males, 17 females) 52.6 ± 12.6 years	No effects on blood pressure	[33]

↑ increase. ↓ decrease. * Significantly different from baseline. ** Significantly different from control. ^a^ Significantly different in African American subjects. HF: high-flavanol; MF: medium-flavanol; LF: low-flavanol; TG: triglycerides; HDL; high-density lipoproteins; SBP: systolic blood pressure; DBP: diastolic blood pressure; FMD: flow-mediated dilatation; NO: nitric oxide; BP: blood pressure.

Table 3 shows the effects of flavanol-rich cocoa on visual and cognitive functions. A study carried out on ten healthy physically active men showed that a single dose of an HF cocoa drink (563 mg flavanols) significantly increased pre-exercise blood glucose levels compared to a low-flavanol (LF) drink, which may have positive effects by improving executive function (effectively enhancing exercise-induced cognitive function improvement) [34]. Another study conducted in 18 healthy older adults (aged 50–65 years) reported a significant increase in cerebral blood flow after a single dose of an HF (494 mg) cocoa drink compared to an LF cocoa drink [35]. Similarly, the consumption of an HF (900 mg) cocoa drink for one week significantly increased cerebral blood flow from baseline in 34 healthy elderly subjects; however, there was no difference compared to the consumption of a cocoa drink low in flavanols [36]. Consumption of an HF cocoa drink for 5 days also significantly increased cerebral blood flow from baseline in young females (18–30 years), but there was no difference compared to an LF drink [37]. Another study carried out in elderly subjects (68.3 ± 3 years) reported a significant increase in serum brain-derived neurotrophic factor (BDNF) after the consumption of an HF cocoa drink (494 mg of flavanols) for 12 weeks compared to an LF cocoa drink, correlating with improvements in global cognitive performance [38]. These results are in line with those of Mastroiacovo et al. [39], who found that consumption of a cocoa drink containing 520–990 mg of flavanols for 8 weeks significantly improved cognitive function in elderly people (69.6 years). In a similar way, a single dose of dark chocolate containing 720 mg of flavanols significantly improved cognitive function in healthy young subjects compared to white chocolate. A significant improvement in visual function was also observed [40]. By contrast, no effects on retinal perfusion or subjective visual function were observed in another study in young people following a dose of dark chocolate containing 400 mg of flavanols [41], thus suggesting that 400 mg of flavanols is not enough to improve visual function. Another study conducted in young subjects (mean age 22.2 years) reported an improvement in spatial attention from baseline; however, no effect on temporal attention was found [42].

Table 4 shows the effects of cocoa with HF content on microbiota, UV-induced erythema, and in pregnant women. Consumption of an HF cocoa drink for 4 weeks significantly increased the populations of *Bifidobacterial* and *Lactobacilli,* and decreased that of *Clostridia*. In addition, a significant decrease in plasma triacylglycerol and C-reactive protein was observed [43].

Heinrich et al. [44] studied the effects of consuming a cocoa drink high in flavanols (326 mg) for 6 weeks on UV-induced erythema in healthy women. They observed a significant decrease in erythema and an increase in cutaneous and subcutaneous tissues’ blood flow, as well as in skin density and hydration. Similarly, Mogollon et al. [45] reported a significant increase in skin elasticity after 12 weeks of HF (200 mg) chocolate consumption; however, no effect on UV-induced erythema was observed.

Consumption of chocolate containing 400 mg of flavanols for 12 weeks by pregnant women significantly increased plasma theobromine levels compared to LF chocolate; however, no effects on FMD or BP were observed [46].

### 3.2. Subjects with Disease

The effects of HF cocoa in subjects with disease have also been studied (Table 5). Consumption of an HF (900 mg) cocoa drink for 4 weeks significantly increased FMD and decreased DBP in subjects with end-stage renal disease (mean age 65.5 ± 14 years); however, a significant increase in heart rate (HR) was also observed [47]. Several authors have studied the effects in overweight or obese subjects. A single dose of a cocoa drink containing 701 mg of flavanols significantly increased FMD and attenuated the exercise-induced increase in BP in subjects aged 54.9 ± 2.2 years [48]. Consumption of cocoa products high in flavanols for 4 weeks significantly increased both the basal and peak diameter of the brachial artery, the basal blood flow volume [49] and FMD [50], while also reducing the augmentation index (AIX) of the brachial artery (a measure of arterial stiffness), resulting in improved vasodilation [49,50]. In contrast, no effects on HOMA-IR or insulin-stimulated glucose disposal were observed after consumption of an HF (1218 mg) cocoa drink for 4 weeks by overweight or obese women aged 19–49 years [51]. A longer consumption (12 weeks) was tested in 23 subjects aged 44.9 ± 4.4 years, and showed a significant increase in FMD and a reduction in insulin resistance and DBP compared to an LF drink [52]. Similarly, consumption of HF cocoa drinks for 4 weeks by subjects with type II diabetes mellitus has been found to significantly increase FMD [53,54] and plasma levels of flavanol metabolites [54]. A significant reduction in DBP and the N-terminal pro-B-type natriuretic peptide (NT-proBNP) was also observed after 4 weeks of consumption of an HF (1064 mg) cocoa drink by subjects with chronic heart failure aged 70 ± 10 years, suggesting an improvement in cardiac function [55]. 

On the other hand, consumption of an HF (750 mg) cocoa drink for 4 weeks by subjects with coronary artery disease (64 ± 3 years) showed a significant improvement in endothelial function [56]; however, a lower dose (444 mg) for 6 weeks showed no effect in the same type of patients [57]. In a similar way, a study conducted in postmenopausal hypercholesterolemic women showed an improvement in endothelial function after consuming an HF (446 mg) cocoa drink for 6 weeks, as well as a significant increase in HDL levels [58]. Another study carried out with subjects with hypertension aged 56.6 ± 11.1 years reported that a single dose of an HF (712–1052 mg) cocoa drink significantly reduced SBP, DBP, and mean BP compared to an LF cocoa drink [59]. Two weeks of consuming an HF cocoa drink significantly increased insulin-stimulated changes in brachial artery diameter in hypertensive subjects (mean age 51 years); however, no effect on blood pressure was observed [60]. A significant decrease in HR was also observed after 6 weeks of consumption of HF chocolate in hypertensive men, as compared to LF chocolate [61].

On the other hand, a significant reduction in fatigability from baseline was observed in subjects with Parkinson’s disease and multiple sclerosis after one week [62] and eight weeks [63] of consuming an HF (194 mg) cocoa drink, respectively; however, no significant differences were observed compared to the consumption of an LF (18.36 mg) drink. Contrarily, no effect on fatigability measures was observed after a single dose of an HF (350 mg) cocoa drink in patients with multiple sclerosis [64], suggesting that a single dose is not enough to produce benefits.

## 4. Discussion

Many components of cocoa such as theobromine and minerals such as magnesium, copper, potassium, and calcium may benefit health. In this regard, some studies have highlighted the potential of theobromine as a protector against cancer, inflammation, and cardiovascular disease [65,66]. Dietary magnesium, copper, potassium and calcium can reduce the risk of hypertension and atherosclerosis [67]. For caffeine, both beneficial and harmful effects on several physiological systems have been described, but chocolate and cocoa are not primary sources of caffeine intake [68]. However, cocoa and dark chocolate’s long-recognized benefits for health have been attributed to the potent antioxidant activity of cocoa polyphenols (mainly flavanols).

Based on the results of the present review, it appears that chronic consumption of cocoa products with a high flavanol content in doses ranging from 45.3 mg/d to 1078 mg/d is more beneficial than a single dose. Beneficial effects, mainly on cardiovascular and cognitive function, have been observed, although positive effects on skin and microbiota have also been observed. Overall, there appear not to be significant differences in the reviewed effects between men and women, except in one study which reported a greater effect in women [46]. Regarding improvements in cognitive function, these have mainly been observed in older subjects [32,33,35,36]. Contrarily, no significant age-related differences in effects on cardiovascular health have been found. There also seems to be no difference in effect between healthy and diseased subjects.

Several reviews have highlighted some of the mechanisms of action of cocoa flavanols. Cocoa flavanols directly neutralize free radicals, reduce levels of reactive oxygen species, inhibit pro-oxidant enzymes and upregulate antioxidant defences [1,67]. In addition to their antioxidant actions, these compounds benefit the cardiovascular system by stimulating NO-mediated vasodilation (which results in reduced systemic blood pressure and increased arterial elasticity), have anti-inflammatory properties, reduce insulin resistance (thus lowering the circulating levels of glucose), and enhance the growth of beneficial gut microbiota [1,3,10,11]. In addition, NO acts in combination with BDNF to modulate neural progenitor cell growth and synaptic metabolism for the sustained adequacy of cognitive functions. Interestingly, NO release at the level of the thalamus contributes to the proper functioning of the neurovascular unit through increased blood flow and volume in the context of the brain. On the other hand, diets rich in bioactive compounds, such as polyphenols, induce adult hippocampal neurogenesis (AHN) by increasing synaptic plasticity and promoting long-term hippocampal potentiation, as well as enhancing learning and memory. In addition, they have been shown to increase the expression of two factors closely related to hippocampal neurogenesis: BDNF and the phosphorylated cyclic AMP-response element DNA-binding protein (pCREB) [69]. Cocoa flavanols may also exert beneficial effects through the inhibition of NADPH oxidase, thereby increasing NO levels [1]. Down-regulation of NF-κB and MAPK has also been observed after cocoa consumption, thus suggesting an inhibitory effect on the release of pro-inflammatory cytokines [70].

Unfortunately, the technological process related to the processing of cocoa beans for the manufacture of cocoa products affects the flavanol profile [9] in a qualitative and quantitative way. For instance, high temperatures and roasting times lead to the loss of bioactive compounds of interest, such as flavanols [71,72]. The preservation of polyphenols during cocoa manufacturing is known to be important for their beneficial effects on human health. Therefore, several studies have been carried out to develop procedures to reduce the loss of these compounds [73,74,75]. González-Barrio et al. [73] made a new formulation of dark chocolate that included 15% cocoa powder enriched with polyphenols; it had greatly increased antioxidant properties compared to conventional dark chocolate.

According to the studies included in the present review, for healthy subjects, the necessary daily dose of cocoa powder to reduce oxidative stress would be 4.45–10.63 g, to reduce cardiovascular risk factors, 1.13–22.95 g, and to improve cognitive function, 4.30–24.75 g. For subjects with metabolic disease, the dose needed to reduce cardiovascular risk factors would be 11.15–26.95 g of cocoa powder, and to reduce fatigue in people with Parkinson’s or multiple sclerosis, the dose would be 4.85–8.75 g of cocoa powder. In view of the aforementioned, and considering that one tablespoon of cocoa powder is equivalent to approximately 10 g, fortification with flavanols could be effective in order to enhance the beneficial effects of cocoa while maintaining a lower consumption amount. The dosage of other cocoa products (such as chocolate, cocoa bars or cocoa drinks) will depend on the percentage of cocoa they contain.

It should be noted that despite the promising positive effects of consumption of HF cocoa, more evidence (using the same dosage and trial protocols) is necessary to establish a real cause and effect relationship between HF cocoa intake and the described health benefits. In addition, due to the wide variation in the content of phenolic compounds in cocoa-derived products, it is essential to determine the concentration of these compounds in the foods used before conducting intervention studies.

## 5. Conclusions

A limited number of studies have been carried out so far. Together with a lack of standardized research and the variety of methodologies used, as well as differences in the duration of the interventions and doses used, the age of the subjects, and their physiological or pathological conditions, is difficult to draw comparisons between them. However, the beneficial effects of consuming cocoa rich in flavanols, especially on cardiovascular health and cognitive function, have been observed. Although the beneficial effects of cocoa have been demonstrated, it should be noted that some cocoa-derived products contain added sugars and additional fats that could have harmful effects, so consumption of such products should be occasional and moderate. It is also for this reason that the fortification of such products with cocoa flavanols could be effective in enhancing their beneficial effects whilst maintaining a lower level of consumption. It may also be appropriate to fortify other foods with cocoa flavanols so that the population can consume these compounds in greater quantities and benefit from their health effects.

## Figures and Tables

**Table 1:** Effects on exercise performance and oxidative stress in healthy subjects.

Dose	Duration	Subjects	Effects	Ref
HF cocoa (425 mg) or maltodextrin	weeks	44 male endurance athletes 34.5 ± 7.5 years	↑* plasma follistatin ↓* body fat ↓* plasma leptin	[17]
HF (1060 mg), MF (746 mg), LF (406 mg), or control (CON) (88 mg) chocolate bar Cross-over	Single dose	15 healthy subjects (10 males, 5 females) 30 ± 7 years	No effects on oxygen consumption respiratory exchange ratio or (HR)	[18]
HF (425 mg) cocoa or maltodextrin	10 weeks	56 male endurance athletes 35.8 ± 8.1 years	↓** oxidative stress No effect on aerobic capacity or exercise performance	[19]
HF (187 mg) or LF (14 mg) cocoa drink Cross-over	Single dose	20 healthy males 20–40 years	↓** oxidative stress	[20]
HF (25 mg/kg body weigh), MF (18.78 mg/kg) or LF (12.5 mg/kg) cocoa drink Cross-over	Single dose	8 healthy male subjects 26 ± 2 years	↓* susceptibility to free radical-induced haemolysis (all doses)	[21]

↑ increase. ↓ decrease. * Significantly different from baseline. ** Significantly different from control. HF: high-flavanol; MF: medium-flavanol; LF: low-flavanol; HR: heart rate.

**Table 3:** Effects on brain-related factors and cognitive function in healthy subjects.

Dose	Duration	Subjects	Effects	Ref
HF (494 mg) or LF (23 mg) cocoa drink Cross-over	Single dose	18 healthy older adults (8 males, 10 females) 61 years	↑** cerebral blood flow	[35]
HF (900 mg/d) or LF (36 mg/d) cocoa drink	1 weeks	34 healthy elderly subjects (16 males, 18 females) 72 ± 6 years	↑* blood flow velocity (MFV) in the middle cerebral artery	[36]
HF (172 mg) or LF (13 mg) cocoa drink	5 days	16 healthy young females 18–30 y	↑* cerebral blood flow	[37]
HF (494 mg) or LF (23 mg) cocoa drink	12 weeks	40 healthy older subjects (22 males, 18 females) 68.3 ± 3 years	↑** serum BDNF levels	[38]
HF (990 mg), MF (520 mg) or LF (45 mg) cocoa drink	8 weeks	90 healthy elderly subjects (37 males, 53 females) 69.6 years	↑^a^ cognitive function ↓^a^ insulin resistance, BP, and lipid peroxidation	[39]
HF (720 mg) dark chocolate or white chocolate Cross-over	Single dose	30 healthy subjects (8 males, 22 females) 18–25 years	↑** visual function ↑** cognitive function	[40]
HF (400 mg) dark chocolate or milk chocolate (5 mg) Cross-over	Single dose	22 healthy subjects (9 males, 13 females) 27.3 ± 11.1 years	No effects on retinal perfusion or subjective visual function	[41]
HF (747 mg), MF (520 mg) or LF (374 mg) cocoa drink	Single dose	48 healthy subjects (24 males, 24 females) 22.2 years	↑* spatial attention No effects on temporal attention	[42]

↑ increase. ↓ decrease. * Significantly different from baseline. ** Significantly different from control. ^a^ Significantly different in the high- and middle-cocoa groups with respect to the low-cocoa group. HF: high-flavanol; MF: medium-flavanol; LF: low-flavanol; BDNF: brain-derived neurotrophic factor; BP: blood pressure.

**Table 4:** Other effects in healthy subjects.

Dose	Duration	Subjects	Effects	Ref
HF (494 mg) cocoa drink or placebo (29 mg) Cross-over	4 weeks	22 healthy subjects (12 males, 10 females) 30.2 ± 11.8 years	↑** Bifidobacterial and lactobacilli populations ↓** Clostridia population ↓** Plasma triacylglycerol and C-reactive protein	[43]
HF (326 mg/d) or LF (27 mg/d) cocoa drink	6 weeks	24 healthy women 18–65 years	↓** UV-induced erythema ↑** blood flow of cutaneous and subcutaneous tissues, skin density and skin hydration	[44]
HF (200 mg) or LF (<30 mg) chocolate	12 weeks	74 healthy women 39.5 ± 13.1 years	↑** net skin elasticity No effects on UV-induced erythema	[45]
HF (400 mg) or LF (<60 mg) chocolate	12 weeks	44 healthy pregnant women 29.2 ± 3.4 years	↑** Plasma levels of theobromine No effects on FMD or BP	[46]

↑ increase. ↓ decrease. ** Significantly different from control. HF: high-flavanol; LF: low-flavanol; UV: ultraviolet; FMD: flow-mediated dilatation; BP: blood pressure.

**Table 5:** Effects in subjects with disease.

Dose	Duration	Subjects	Effects	Ref
HF (900 mg) cocoa drink or non-containing placebo	4 weeks	52 subjects with end stage renal disease (38 males, 14 females) 65.5 ± 14 years	↑** FMD ↓** DBP ↑** heart rate	[47]
HF (701 mg) or LF (22 mg) cocoa drink Cross-over	Single dose	21 subjects with overweight or obesity (13 males, 8 females 54.9 ± 2.2 years	↓** Exercise-induced increases in BP ↑** FMD	[48]
HF (814 mg) or LF (3 mg) dark chocolate and cocoa drink Cross-over	4 weeks	30 subjects with overweight (15 males, 15 females) 51.7 ± 1.2 years	↑** basal diameter and peak diameter of the brachial artery ↑** basal blood flow volume ↓^a^ AIX No effects on fasting blood measures	[49]
HF (1078 mg) or LF (259 mg) chocolate Cross-over	4 weeks	44 men with overweight 63 ± 5 years	↑* FMD ↓* AIX ↓* leukocyte cell count and adhesion	[50]
HF (1218 mg/d) or LF (26 mg/d) cocoa drink	4 weeks	32 females with overweight or obesity 33.4 ± 10.2 years	No effects on HOMA-IR or insulin-stimulated glucose disposal	[51]
HF (902 mg) or LF (36 mg) cocoa drink	12 weeks	23 subjects with overweight or obesity (7 males, 16 females) 44.9 ± 4.4 years	↑** FMD ↓** insulin resistance ↓** DBP and mean arterial BP	[52]
HF (963 mg/d) or LF (75 mg/d) cocoa drink	4 weeks	44 subjects with type II diabetes 50–80 years	↑** plasma flavanol metabolite levels ↑** FMD	[54]
HF (963 mg/d) or LF (75 mg/d) cocoa drink Cross-over	4 weeks	51 subjects with type II diabetes (20 males, 31 females) 63.8± 8.5 years	↑* FMD	[53]
HF (1064 mg) or LF (88 mg) dark chocolate	4 weeks	24 subjects with chronic heart failure (20 males, 4 females) 70 ± 10 years	↓** NT-proBNP ↓** DBP	[55]
HF (750 mg) or LF (18 mg) cocoa drink Cross-over	4 weeks	16 subjects with coronary artery disease (13 males, 3 females) 64 ± 3 years	↓** levels of endothelial microparticles ↑** endothelial function	[56]
HF (444 mg/d) chocolate bar and cocoa drink or placebo (16.9 mg/d)	6 weeks	40 subjects with coronary artery disease (30 males, 10 females) 61 ± 8 years	No effects on brachial artery FMD or systemic arterial compliance No effects on forearm blood flow	[57]
HF (446 mg) or LF (43 mg) cocoa drink	6 weeks	32 postmenopausal hypercholesterolemic women 56.6 ± 2.0 years	↓** sVCAM-1 ↑** HDL ↓^b^ SBP and DBP	[58]
HF (712–1052 mg), MF (372 mg) or LF (33 mg) cocoa drink	Single dose	32 men and 20 postmenopausal women with untreated mild hypertension 56.6 ± 11.1 years	↓** SBP, DBP and mean arterial BP	[59]
HF (902 mg) cocoa drink or placebo drink (28 mg) Cross-over	2 weeks	20 subjects with hypertension (8 males, 12 females) 51.0 ± 1.5 years	↑** Insulin-stimulated changes in brachial artery diameter No effects on BP or insulin resistance	[60]
HF (1064 mg) or LF (88 mg) dark chocolate Cross-over	6 weeks	32 men with hypertension 55.4 ± 1.5 years	↓** HR increase	[61]
200 mL HF (194 mg) or LF (18.36 mg) cocoa drink	1 week	30 subjects with Parkinson (18 males, 12 females) 64.2 ± 11.6 years	↓ fatigability *	[62]
HF (194 mg) or LF (18.36 mg) cocoa drink Crossover	8 weeks	40 subjects with multiple sclerosis (10 males, 30 females) 43.5 ± 9.5 years	↓ fatigability *	[63]
HF (350 mg) or LF (120 mg) cocoa drink Crossover	Single dose	12 subjects with multiple sclerosis (2 males, 10 females) 54 ± 10.56 years	↓ self-reported fatigue ↓ activity during sleeping ↑ physical activity ↓ glycaemic response No effect on fatigability measures	[64]

↑ increase. ↓ decrease. * Significantly different from baseline. ** Significantly different from control. ^a^ Significantly different only in women. ^b^ Significantly different in the low-cocoa group respect baseline. HF: high-flavanol; MF: medium-flavanol; LF: low-flavanol; FMD: flow-mediated dilatation; DBP: diastolic blood pressure; BP: blood pressure; AIX: augmentation index; NT-proBNP: N-terminal pro-B-type natriuretic peptide; SBP: systolic blood pressure; HR: heart rate.

## Abbreviations

AHN	Adult hippocampal neurogenesis
AIX	Augmentation index
BDNF	Brain-derived neurotrophic factor
BP	Blood pressure
DBP	Diastolic blood pressure
FMD	Flow-mediated dilatation
HF	High-flavanol
LF	Low-flavanol
NO	Nitric oxide
NT-proBNP	N-terminal pro-B-type natriuretic peptide
pCREB	Phosphorylated cyclic AMP-response element DNA-binding protein
PCs	Phenolic compounds
SBP	Systolic blood pressure
